# The Interferon-Induced Exonuclease ISG20 Exerts Antiviral Activity through Upregulation of Type I Interferon Response Proteins

**DOI:** 10.1128/mSphere.00209-18

**Published:** 2018-09-19

**Authors:** Christopher M. Weiss, Derek W. Trobaugh, Chengqun Sun, Tiffany M. Lucas, Michael S. Diamond, Kate D. Ryman, William B. Klimstra

**Affiliations:** a Department of Microbiology and Molecular Genetics, University of Pittsburgh, Pittsburgh, Pennsylvania, USA; b Center for Vaccine Research, University of Pittsburgh, Pittsburgh, Pennsylvania, USA; c Department of Immunology, University of Pittsburgh, Pittsburgh, Pennsylvania, USA; d Department of Pathology and Immunology, Washington University School of Medicine, Saint Louis, Missouri, USA; e Department of Medicine, Washington University School of Medicine, Saint Louis, Missouri, USA; f Andrew M. and Jane M. Bursky Center for Human Immunology and Immunotherapy Programs, Washington University School of Medicine, Saint Louis, Missouri, USA; UT Southwestern Medical Center

**Keywords:** alphavirus, host-pathogen interactions, innate immunity, interferon-stimulated gene, virus-host interactions

## Abstract

Type I interferon (IFN)-stimulated genes (ISGs) have critical roles in inhibiting virus replication and dissemination. Despite advances in understanding the molecular basis of ISG restriction, the antiviral mechanisms of many remain unclear. The 20-kDa ISG ISG20 is a nuclear 3′–5′ exonuclease with preference for single-stranded RNA (ssRNA) and has been implicated in the IFN-mediated restriction of several RNA viruses. Although the exonuclease activity of ISG20 has been shown to degrade viral RNA *in vitro*, evidence has yet to be presented that virus inhibition in cells requires this activity. Here, we utilized a combination of an inducible, ectopic expression system and newly generated *Isg20*^−/−^ mice to investigate mechanisms and consequences of ISG20-mediated restriction. Ectopically expressed ISG20 localized primarily to Cajal bodies in the nucleus and restricted replication of chikungunya and Venezuelan equine encephalitis viruses. Although restriction by ISG20 was associated with inhibition of translation of infecting genomic RNA, degradation of viral RNAs was not observed. Instead, translation inhibition of viral RNA was associated with ISG20-induced upregulation of over 100 other genes, many of which encode known antiviral effectors. ISG20 modulated the production of IFIT1, an ISG that suppresses translation of alphavirus RNAs. Consistent with this observation, the pathogenicity of IFIT1-sensitive alphaviruses was increased in *Isg20*^−/−^ mice compared to that of wild-type viruses but not in cells ectopically expressing ISG20. Our findings establish an indirect role for ISG20 in the early restriction of RNA virus replication by regulating expression of other ISGs that inhibit translation and possibly other activities in the replication cycle.

**IMPORTANCE** The host immune responses to infection lead to the production of type I interferon (IFN), and the upregulation of interferon-stimulated genes (ISGs) reduces virus replication and virus dissemination within a host. Ectopic expression of the interferon-induced 20-kDa exonuclease ISG20 suppressed replication of chikungunya virus and Venezuelan equine encephalitis virus, two mosquito-vectored RNA alphaviruses. Since the replication of alphavirus genomes occurs exclusively in the cytoplasm, the mechanism of nucleus-localized ISG20 inhibition of replication is unclear. In this study, we determined that ISG20 acts as a master regulator of over 100 genes, many of which are ISGs. Specifically, ISG20 upregulated IFIT1 genes and inhibited translation of the alphavirus genome. Furthermore, IFIT1-sensitive alphavirus replication was increased in *Isg20*^−/−^ mice compared to the replication of wild-type viruses but not in cells ectopically expressing ISG20. We propose that ISG20 acts as an indirect regulator of RNA virus replication in the cytoplasm through the upregulation of many other ISGs.

## Abstract (precis)

The host immune responses to infection lead to the production of type I interferon (IFN), and the upregulation of interferon-stimulated genes (ISGs) reduces virus replication and virus dissemination within a host. Ectopic expression of the interferon-induced 20-kDa exonuclease ISG20 suppressed replication of chikungunya virus and Venezuelan equine encephalitis virus, two mosquito-vectored RNA alphaviruses. Since the replication of alphavirus genomes occurs exclusively in the cytoplasm, the mechanism of nucleus-localized ISG20 inhibition of replication is unclear. In this study, we determined that ISG20 acts as a master regulator of over 100 genes, many of which are ISGs. Specifically, ISG20 upregulated IFIT1 genes and inhibited translation of the alphavirus genome. Furthermore, IFIT1-sensitive alphavirus replication was increased in *Isg20*^−/−^ mice compared to the replication of wild-type viruses but not in cells ectopically expressing ISG20. We propose that ISG20 acts as an indirect regulator of RNA virus replication in the cytoplasm through the upregulation of many other ISGs.

## INTRODUCTION

Innate immune responses provide a first line of defense against many pathogens and act to stimulate adaptive immune responses, which in turn help to clear infections and generate lasting immunological memory. Stimuli from invading viruses trigger pathogen recognition receptors that induce antiviral gene regulatory programs in cell-intrinsic (e.g., antiviral effector proteins) and cell-extrinsic (e.g., cytokines) manners. The most widely studied antiviral innate immune cytokine produced by this process, type I interferon (IFN), signals through its heterodimeric receptor (IFNAR1/IFNAR2) on infected and uninfected cells, activating the IFN-stimulated gene factor 3 complex (ISGF3) to transcribe a targeted and yet large set of interferon-stimulated genes (ISG). These ISGs control a variety of antimicrobial functions by indirectly or directly limiting replication of the invading pathogen (1).

Alphaviruses are small, single-stranded RNA viruses (ssRNA) that are typically spread via the bite of an arthropod vector. These include the arthritogenic viruses such as chikungunya virus (CHIKV), Sindbis virus (SINV), and Ross River virus (RRV) and the encephalitogenic viruses such as Eastern equine encephalitis virus (EEEV), Western equine encephalitis virus (WEEV), and Venezuelan equine encephalitis virus (VEEV). This genus of viruses includes emerging pathogens as well as one of the most pathogenic RNA viruses (EEEV) for humans. Several studies have revealed a role for individual IFN-upregulated effector proteins in controlling alphavirus infection (2–9). One ISG with potent antiviral activity *in vitro* against alphaviruses is the 20-kDa exonuclease interferon-stimulated gene 20 (ISG20) (3). ISG20 is a member of the RNA-specific DEDD family of 3′-5′ exonucleases that is localized predominantly in Cajal bodies, which are dense subnuclear structures of protein and RNA (10–12). ISG20 has been shown to restrict infection of multiple RNA and DNA viruses (3, 13–20). Due to the capacity of ISG20 to degrade RNA nonspecifically *in vitro* and a more limited effect on DNA viruses, it has been suggested that ISG20 functions as an antiviral by directly degrading viral genomic RNA. However, this presumed activity has not been demonstrated in cells where ISG20 and many viral RNAs localize to different subcellular compartments. For example, the alphavirus genome replicates entirely within the cytoplasm of infected cells and would not typically encounter a nucleus-localized protein.

We evaluated the antiviral activity of ISG20 and its effect on alphavirus replication. Ectopic expression of ISG20 in fibroblasts potently restricted the replication of multiple alphaviruses through a block in viral translation but, unexpectedly, did not accelerate the degradation of viral RNA. Nonetheless, the replication-blocking activity was dependent on the presence of an intact ISG20 exonuclease domain. In the absence of viral infection or IFN gene induction, ectopic expression of ISG20, but not a nuclease domain mutant, induced expression of a suite of genes, many of which have described or predicted antiviral activity. IFIT1 was consistently among the genes that were most highly upregulated by ISG20 overexpression. IFIT1 has been shown to play a prominent role in host cell recognition of non-self-RNAs and suppression of viral mRNA translation (6). Although *Isg20*^−/−^ mice were not more susceptible to infection with wild-type (WT) pathogenic strains of CHIKV or VEEV, an IFIT1-sensitive mutant of VEEV was more virulent in *Isg20*^−/−^ mice and replicated to higher levels in some, but not all, *Isg20*^−/−^ primary cells than in the WT counterparts. Taken together, our data suggest a role for ISG20 as a regulator of steady-state and IFN-induced antiviral activity in selected cell types.

(Portions of this work were uploaded to the D-Scholarship Institutional Repository at the University of Pittsburgh as part of dissertation work [C. M. Weiss].)

## RESULTS

### Murine ISG20 expression restricts alphavirus replication.

**FIG 1:**
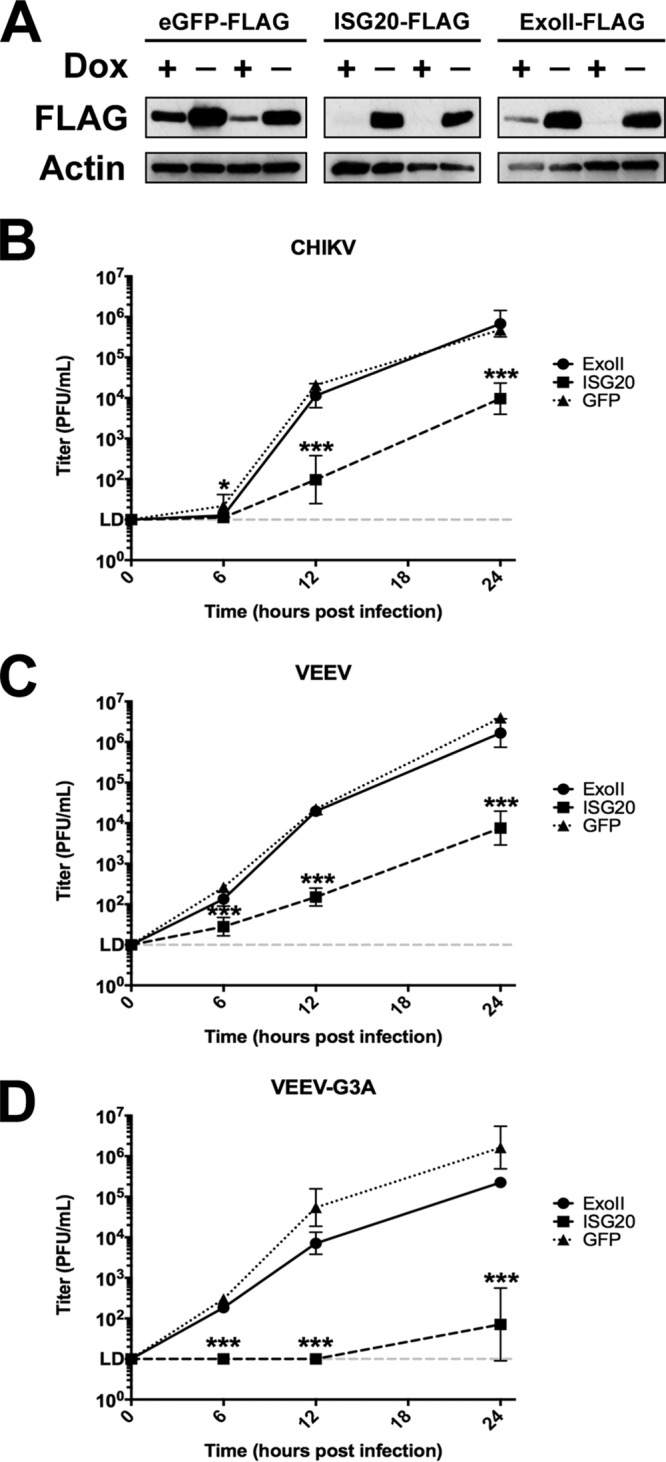
Inducible expression of murine ISG20 suppresses alphavirus replication. (A) Two clones of tet-off MEFs expressing FLAG-tagged eGFP, murine ISG20, or the exonuclease domain mutant murine ISG20^D94G^ (ExoII) were induced for 72 h and then immunoblotted for the FLAG-tagged protein. DOX, doxycycline. (B to D) Induced tet-off MEFs were infected with (B) WT CHIKV, (C) WT VEEV, or (D) VEEV-G3A (MOI of 0.1). Replicated virus levels were measured in cell supernatants by BHK-21 plaque assay. *, *P* < 0.5; ***, *P* < 0.001 (2-way ANOVA with Holm-Sidak *post hoc* analysis against eGFP control) (*n* = 6, 2 independent experiments).

We first evaluated the antiviral activity of murine ISG20 against CHIKV and VEEV, utilizing a stable, inducible expression system in the absence of type I IFN treatment. Two separately derived clonal cell lines were derived using tetracycline-inducible (tet-off) murine embryonic fibroblasts (MEFs) expressing C-terminally FLAG-tagged murine ISG20; a mutant, ISG20^D94G^, that is homologous to a human ISG20 mutant with disrupted exonuclease activity (Exo II) (11); and enhanced green fluorescent protein (eGFP) and firefly luciferase (fLuc) as controls (3). We confirmed the expression of each protein by Western blotting for the FLAG epitope tag (Fig. 1A). Cellular localization of ectopically expressed ISG20 was consistent with the published literature, forming dense nuclear puncta in confocal micrographs (see Fig. S1A in the supplemental material), and this localization did not change with virus infection (data not shown). To assess antiviral restriction by ISG20, tet-off cells were induced for 72 h and infected with WT CHIKV or VEEV, as well as with VEEV-G3A, which encodes an attenuating mutation in the 5′nontranslated region that was acquired during cell adaptation of the TC83 vaccine strain of VEEV. This mutation diminishes virulence in mice due to enhanced sensitivity to the antiviral effector activity of IFIT1 (6). Ectopic expression of ISG20 in MEFs reduced infection of WT CHIKV and VEEV by approximately 100-fold at 12 and 24 h postinfection (h.p.i.) (Fig. 1B and C). However, replication in MEFs expressing the ISG20 ExoII mutant was similar to that seen with cells expressing the GFP control, confirming that the exonuclease of ISG20 was essential for antialphavirus activity. VEEV-G3A was more sensitive to the antiviral effects of ISG20, as infection was restricted completely at 6 and 12 h.p.i. by ISG20 expression and was 1,000-fold lower at 24 h.p.i. than that seen with MEFs expressing the GFP or ExoII mutant (Fig. 1D).

10.1128/mSphere.00209-18.1 FIG S1 Expression of ISG20 reduces the number of productively infected cells. (A) tet-off MEFs expressing murine ISG20-FLAG or murine ISG20^D94G^-FLAG (ExoII) were grown on glass cover slips and induced for 72 h. ISG20 was detected by the use of FITC-conjugate anti-FLAG (M1) monoclonal antibody, and localization was determined by confocal microscopy. Images represent an averaged z-stack projection at 100× objective magnification. (B) tet-off MEFs expressing the indicated protein were infected with CHIKV-TaV-GFP or VEEV-TaV-eGFP fluorescent reporter virus (MOI of 1). Percentages of tet-off MEFs infected are represented. (C) Representative images of tet-off MEFs infected with CHIKV-GFP or VEEV-GFP. Images were obtained by epifluorescence microscopy at 24 h.p.i. ***, *P *<* *0.001 (2-way ANOVA with Holm-Sidak *post hoc* analysis against eGFP control [B and C]) (*n* = 6, 2 independent experiments). Download FIG S1, JPG file, 2.4 MB. Copyright © 2018 Weiss et al. 2018 Weiss et al. This content is distributed under the terms of the Creative Commons Attribution 4.0 International license.

CHIKV or VEEV expressing GFP as a nonstructural protein 3 (nsP3) fusion (21) was used to determine whether ISG20-dependent restriction was a result of fewer cells initiating infection or represented a uniform reduction in replication efficiency. At 24 h after infection, fluorescence microscopy performed to assess GFP expression revealed a lower percentage of GFP-positive cells (Fig. S1B) and reduced fluorescence intensity (Fig. S1C), suggesting that ISG20 inhibited overall replication efficiency. Again, the ISG20 ExoII mutant did not inhibit CHIKV or VEEV infection as the percentage of infected cells was similar to the percentage of control cells expressing fLuc.

### ISG20 expression blocks viral genome translation.

**FIG 2:**
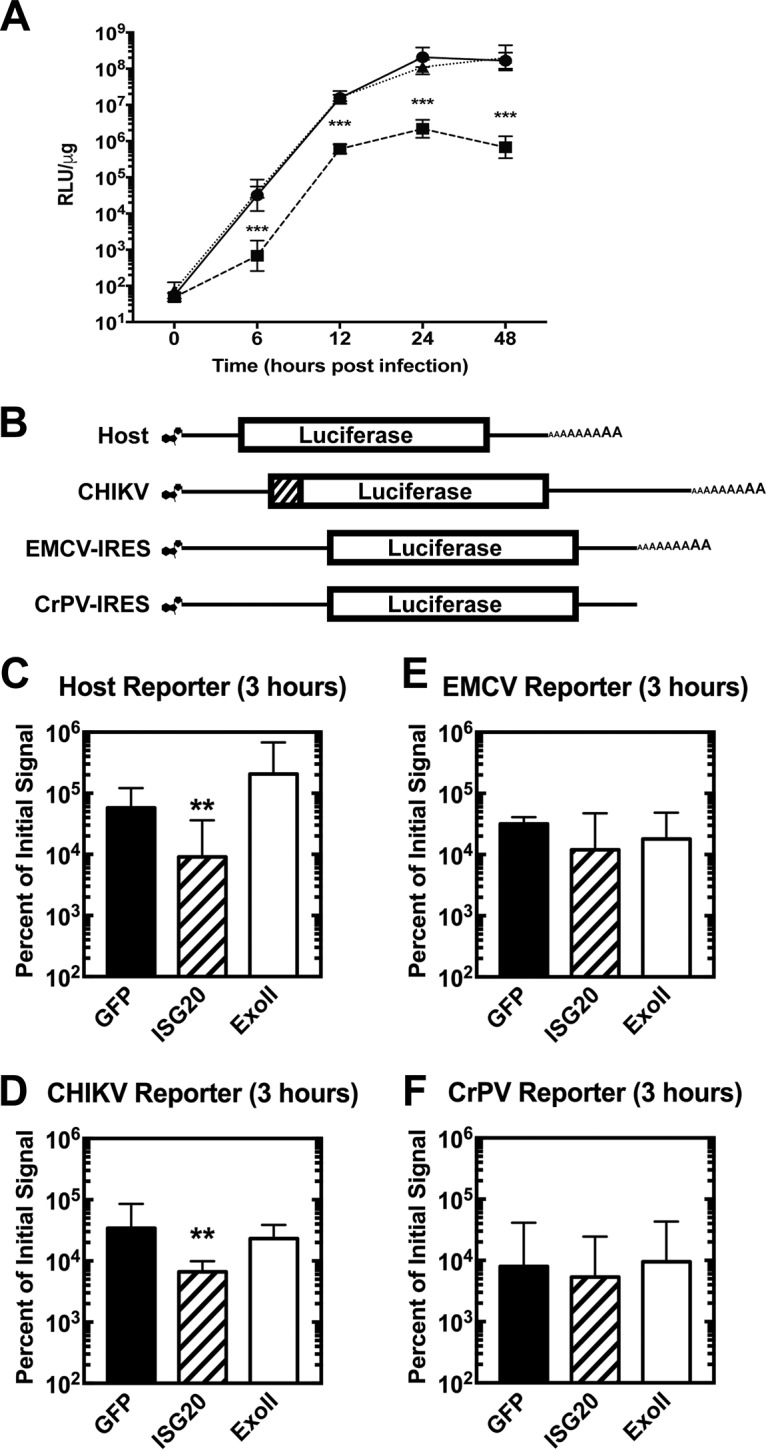
ISG20 suppresses translation of non-IRES-containing capped mRNA. (A) tet-off MEFs overexpressing the indicated protein were infected with CHIKV-nsP3-nLuc nonstructural reporter virus at an MOI of 0.1, and lysates were collected at the indicated time points. Reporter signal data are given as ratios of relative luminescence units normalized to cellular protein levels in micrograms (RLU/μg). ***, *P* < 0.001 (2-way ANOVA with Holm-Sidak *post hoc* analysis against GFP control) (*n* = 6, 2 independent experiments). (B) Schematic representation of type 0 capped and polyadenylated nonreplicating translation reporters. The luciferase gene is flanked by 5′ and 3′ nontranslated regions for the indicated mRNA. The CHIKV reporter also encodes the translational initiation sequences of the nsP1 protein (diagonal lines). (C to F) Induced tet-off MEFs overexpressing the indicated protein were electroporated with 7.5 μg of indicated RNA and then sampled for 4 h. Relative luminescence at 3 h is given as a percentage of the initial signal at 30 min. (C) Host reporter. (D) CHIKV reporter. (E) EMCV reporter. (F) CrPV reporter. **, *P* < 0.01 (2-way ANOVA with Holm-Sidak *post hoc* analysis against eGFP control) (*n* = 6, 2 independent experiments).

To explore the mechanism behind ISG20 suppression of virus replication, we infected tet-off MEFs expressing ISG20, ExoII, or GFP with a CHIKV strain expressing nanoluciferase (nLuc) in nsP3 as a reporter for the translation of the incoming virus genome. The significant reduction in CHIKV nsP3 reporter signal in the presence of ISG20 at early time points (e.g., 6 h postinfection; Fig. 2A) suggested a possible inhibition of the translation of the incoming nonstructural polyprotein. To evaluate this more directly, we tested whether ISG20 could inhibit the translation of incoming 5′ capped RNA using reporters encoding only the 5′ and 3′ nontranslated regions of a generic RNA (host) or the 5′ and 3′ nontranslated regions of CHIKV fused inframe with a firefly luciferase mRNA sequence (Fig. 2B) (22). Transfection of these RNAs into MEFs expressing GFP, ISG20, or ExoII revealed a significant decrease in peak translation of the host RNA (Fig. 2C) and CHIKV reporter RNA (Fig. 2D) when ISG20, but not Exoll, was expressed. Internal ribosomal entry site (IRES) sequences can be utilized by some RNAs to initiate translation without the use of components of the cap-dependent translation machinery (23). The IRES from encephalomyocarditis virus (EMCV) requires some cap-dependent translation initiation factors (IFs) to initiate translation, whereas the IRES used by cricket paralysis virus (CrPV) does not utilize this pathway for translation initiation (23). Notably, transfected EMCV-IRES and CrPV-IRES reporters were unaffected by ISG20 expression (Fig. 2E and F). These data suggest that ISG20 expression blocks only cap-dependent translation initiation rather than inducing global inhibition of protein synthesis in MEFs. These data are similar to results obtained by evaluating the effects of interferon alpha-beta (IFN-α/β) treatment on translation of virus reporter RNAs in cells (24).

### ISG20 expression does not accelerate decay of the CHIKV RNA 3′ terminus.

**FIG 3:**
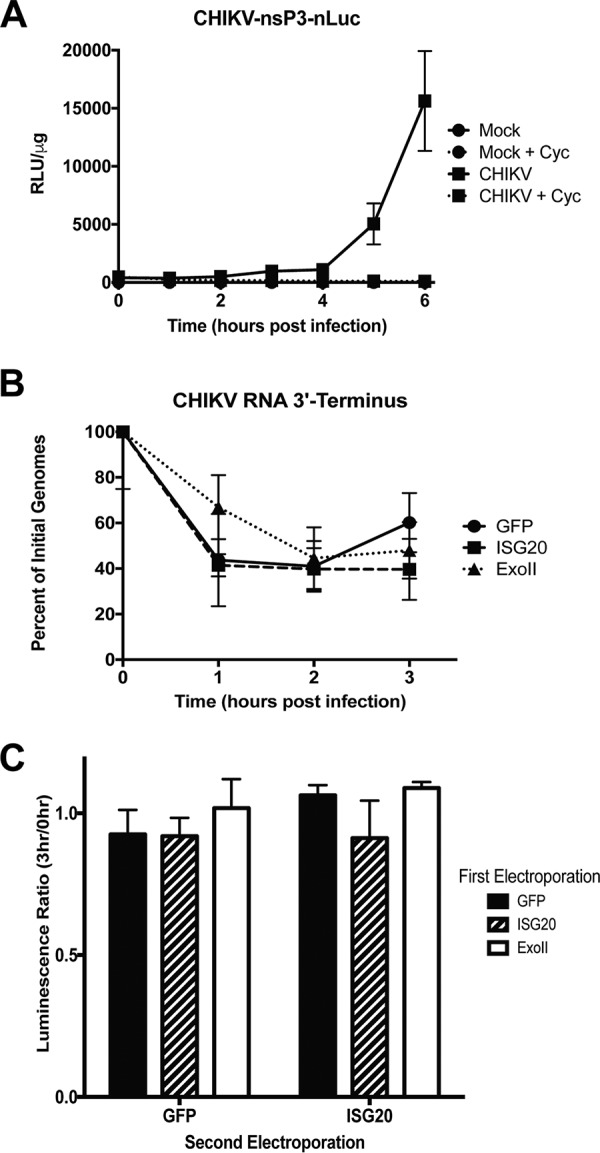
ISG20 expression does not accelerate degradation of CHIKV RNA. (A) MEFs were infected with CHIKV expressing nLuc in frame with the nsP3 nonstructural protein at an MOI of 5 and then treated with 0 or 10 μM cycloheximide (Cyc) to block translation and, ultimately, replication of CHIKV RNA. (B) MEFs were infected with CHIKV-LR (MOI = 5) and then treated with 10 μM cycloheximide. RNA was collected at the indicated time p.i. and analyzed by qRT-PCR for the CHIKV 3′ terminus. (B) Data represent results of 2-way ANOVA with Holm-Sidak *post hoc* analysis against eGFP control. (*n* = 6, 2 independent experiments; data were not statistically significant). (C) RNA isolated from ISG20 MEFs is functional. The CHIKV-translation reporter was isolated from tet-off MEFs overexpressing GFP, ISG20, or ExoII mutant, retransfected into MEFs expressing either GFP or ISG20 for 3 h, and assayed for firefly luciferase reporter activity, and luciferase activity data from second transfections are given as ratios of firefly luciferase signals from h 3 versus h 0 samples taken from first transfection. Data represent results of ANOVA with Dunnett’s *post hoc* analysis against GFP control (*n* = 6, 2 independent experiments; data were not statistically significant).

ISG20 was previously characterized as a 3′–5′ exoribonuclease capable of degrading nonspecific RNA targets in a processive manner *in vitro* (11). Testing the effects of ISG20 expression on degradation of viral RNA requires introduction of RNA into the cell cytoplasm and inhibition of RNA replication. Cycloheximide, a potent global inhibitor of translation, completely blocks translation of the CHIKV RNA (Fig. 3A), which inhibits virus replication. Induced tet-off MEFs expressing ISG20, ExoII, or control GFP were infected with CHIKV (multiplicity of infection [MOI] of 5) and treated with cycloheximide. Primers for quantitative real-time PCR (qRT-PCR) were designed to target the 3′ terminus of CHIKV to detect the earliest signs of degradation within the 3′ nontranslated region. qRT-PCR analysis revealed no significant differences in the number of CHIKV genomes in the GFP, ISG20, or ExoII tet-off MEFs over time (Fig. 3B), demonstrating that ISG20 expression does not accelerate the decay of CHIKV RNA. This result is similar to our previous results demonstrating that Sindbis virus RNA is not degraded in IFN-treated cells that have upregulated ISGs, including ISG20 (24).

To confirm that ISG20 expression does not degrade CHIKV RNA, we electroporated the CHIKV-translation reporter RNA into cells expressing GFP, ISG20, or ExoII. After 0 h and 3 h, total cellular RNA was harvested and reelectroporated into either GFP-expressing cells or ISG20-expressing cells and analyzed for luciferase activity. If ISG20 were degrading CHIKV RNA, we would expect to observe less translation from the CHIKV RNA isolated from ISG20-expressing cells at 3 h than at 0 h. We observed similar levels of translation activity of the 3 h and 0 h samples (ratio, ∼1) after reelectroporation into GFP control cells lacking ISG20 expression (Fig. 3C), demonstrating that prior exposure to ISG20 did not functionally inhibit the CHIKV translation reporter. Overall, these data demonstrate that ISG20 expression does degrade CHIKV RNA in ISG20-overexpressing MEFs.

### ISG20 expression regulates a focused gene signature.

**FIG 4:**
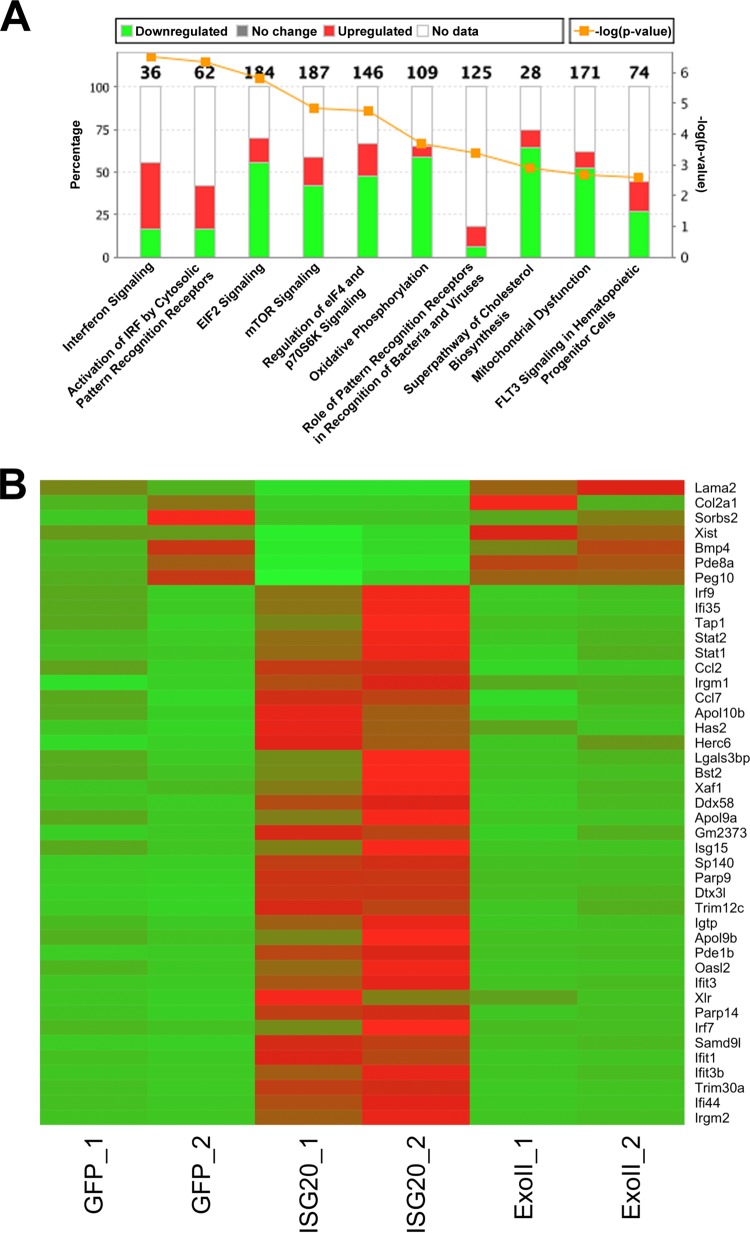
ISG20 expression induces antiviral gene transcription. RNA-seq was performed on two sepearte clones of MEFs expressing eGFP, ISG20, or ExoII. (A) Differential gene expression was analyzed, and upregulated canonical pathways are shown. Numbers on top of each column indicate the total number of genes investigated in each pathway. (B) A heat map of the most significant ISG20-induced genes is shown. Temperature ranges are shown as fragments per kilobase of transcript per million mapped reads (FPKM) from green (low) to red (high). *P* < 0.001 (Cuffdiff 2 gene expression model; false-discovery rate [FDR] = 5%).

**FIG 5:**
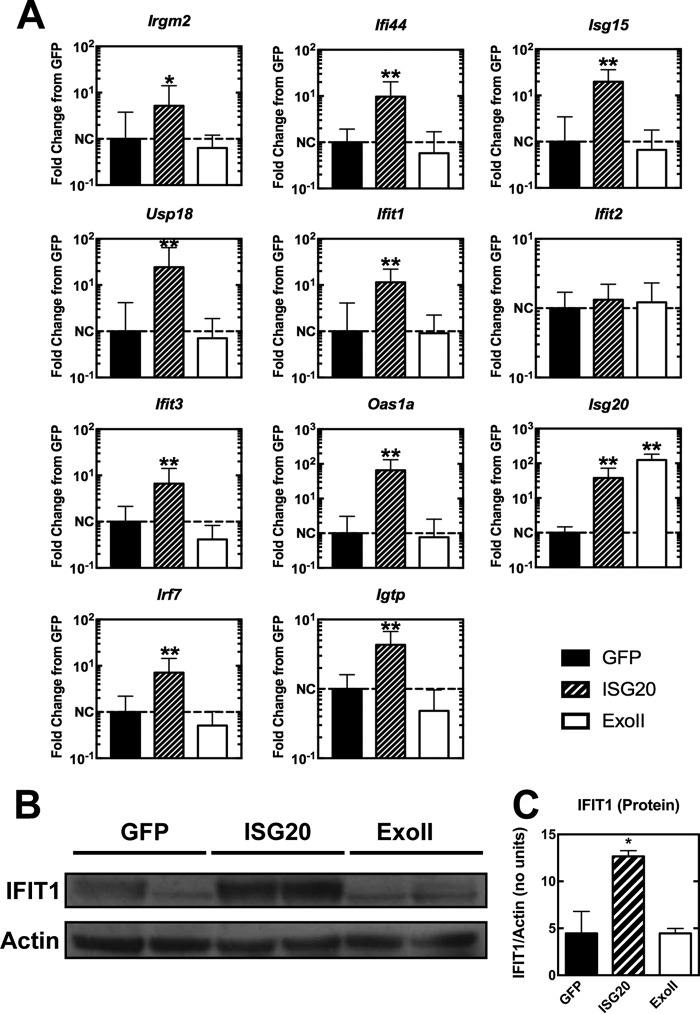
ISG20 induces a subset of antiviral genes. tet-off MEFs expressing eGFP, ISG20, or ExoII mutant were induced for 72 h, and total cellular RNA and protein were collected. (A) A selection of genes shown to be induced by ISG20 overexpression through RNA sequencing were confirmed by qRT-PCR (*n* = 6, 2 independent experiments). NC, no change. (B and C) IFIT1 protein induction was confirmed by Western blotting (B) and quantified (C). (A and C) *, *P* < 0.05; **, *P* < 0.01 (Mann-Whitney test). (For panels B and C, *n* = 2, 2 independent experiments).

Type I IFN signaling and gene induction inhibit alphavirus translation through the actions of multiple gene products (2, 24). Since both alphavirus replication and translation reporter activity were inhibited strongly by ISG20 in the absence of effects on RNA stability, which resembled the effect of IFN-α/β treatment, we transcriptionally profiled cells to determine if antiviral mechanisms or ISGs were altered in the ISG20-expressing cells. We performed RNA sequencing (RNA-seq) on two independently generated clones of the tet-off MEFs expressing GFP, ISG20, or ExoII. Using two clones of each cell type ensured that stable expression of the proteins did not generate an outlier cell population. In the absence of infection, MEFs expressing ISG20 revealed an IFN-like pattern of gene expression even though exogenous IFN was not added. The most highly upregulated genes, which exhibited 5-fold or greater induction compared to both control and ExoII mutant cells, were annotated as antiviral or inducible by type I or II IFN (Fig. 4A). However, IFN-α/β, IFN-γ, or IFN-λ genes were not upregulated significantly by ISG20 expression, as determined by RNA-seq or qRT-PCR (data not shown). Among the top gene targets identified were those encoding several IFN-inducible proteins with tetratricopeptide repeats (*Ifit* family), including *Ifit1* and *Ifit3*, as well as other *Ifit*-like genes (Fig. 4B). Not all of the *Ifit* family genes were upregulated by ISG20 expression; *Ifit2* was an exception. Members of several other gene families were induced, including the ubiquitin-like *Isg15*, the ISG15 E3 ligase, *Herc6*, and the de-ISGylating *Usp18* (Fig. 4B). Upregulated genes also included multiple IFN response pathway transcription factor genes (e.g., *Irf7*, *Irf9*, *Stat1*, and *Stat2*) and the viral RNA-sensing helicase gene *Ddx58* (RIG-I). Network analysis of ISG20 overexpression highlighted a pattern similar to that seen with viral infection or IFN responses but without the induction of primary signaling components of type I, II, or III IFN or their receptors. In addition to the IFN-like responses, the levels of eukaryotic initiation factor 2 (eIF2) signaling, eIF4 signaling, and mTOR signaling, all affecting translation efficiency, were increased in response to ISG20 ectopic expression in MEFs (Fig. 4A). Upregulation of the most significantly induced set of genes from RNA-seq was confirmed by qRT-PCR in ISG20 (Fig. 5A). *Ifit1* was consistently upregulated ∼10-fold over baseline levels in cells ectopically expressing ISG20 compared to the cells expressing GFP and ExoII. The ISG20-mediated upregulation in *Ifit1* mRNA levels also led to increased IFIT1 protein expression in the cells that required a functional ISG20 protein (ExoII) (Fig. 5B and C).

### Gene induction by ISG20 requires IRF3 expression.

**FIG 6:**
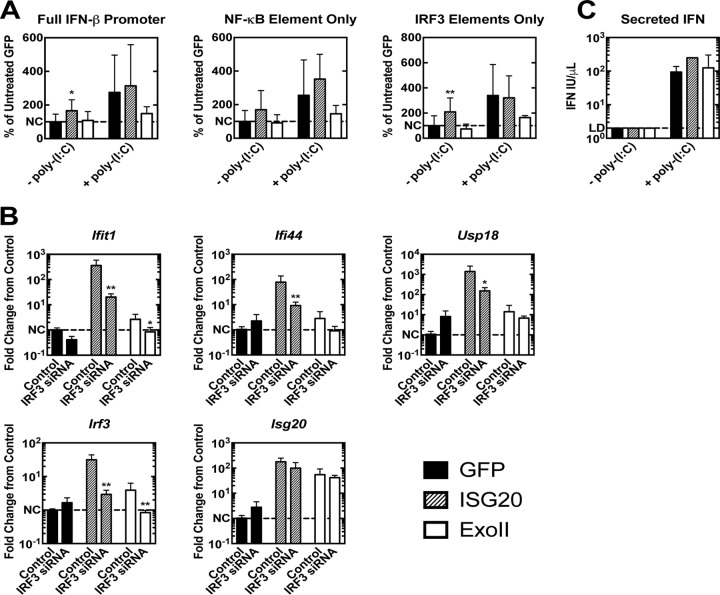
ISG20 expression activates transcriptional pathways involving IRF3. (A) MEFs expressing eGFP, ISG20, and ExoII were induced and transfected with the luciferase driven by the IFN-β promoter (pβLUX) or the IRF3 responsive elements (PRDI/III) or the NF-κB element of the IFN-β promoter (PRDII) along with a SV40 promoter-driven *Renilla* plasmid. Firefly luciferase RLUs were normalized to *Renilla* RLUs, and induction data are given as percentages of untreated GFP cell results. (B) MEFs expressing GFP, ISG20, and ExoII mutant were induced and treated at 24 h with si*Irf3* or siRNA scramble for an additional 48 h. Selected genes were assessed by qRT-PCR, and induction levels are given as fold changes from sham-treated GFP control results. (C) IFN levels were measured in the tet-off MEF cell culture supernatant before and after poly(I·C) lipid transfection. *, *P* < 0.05; **, *P* < 0.01; ***, *P* < 0.001. (A and C) ANOVA with Dunnett’s *post hoc* analysis (*n* = 6, 2 independent experiments). (B) Mann-Whitney test (*n* = 6, 2 independent experiments).

Many of the genes upregulated in the context of ISG20 expression are also upregulated by interferon regulatory factor 3 (IRF3) activation (25). The pathway analysis of the ISG20-regulated genes implicated IRF3 as a prime candidate for regulating the ISG20 response (data not shown). To assess the role of IRF3 in mediating ISG20-dependent induction of ISGs, we transfected luciferase plasmids containing the complete IFN-β promoter or its constituent two IRF3-binding elements or one NF-κB-binding element into our MEFs. ISG20 expression led to a significant increase in luciferase expression in cells transfected with the entire IFN-β promoter as well as in cells transfected with the partial promoters containing only the two IRF3 components (Fig. 6A). Luciferase expression was also increased in ISG20-expressing cells transfected with the NF-κB element alone, suggesting that ISG20 expression may also drive NF-κB promoter activity (Fig. 6A). Poly(I·C) treatment led to the stimulation of the complete IFN-β promoter and its constituent elements in both GFP control cells and the ISG20-expressing cells, demonstrating that IFN treatment can also lead to NF-κB and IRF3 promoter activity in the absence of ISG20 overexpression (Fig. 6A). Expression of the Exoll ISG20 mutant did not drive promoter activity of the complete IFN-β promoter or its constituent elements, and the results actually trended toward reduced promoter activation after poly(I·C) treatment (Fig. 6A). Furthermore, small interfering RNA (siRNA) knockdown of IRF3 in tet-off MEFs expressing ISG20 revealed a significant reduction in ISG20-modulated gene upregulation versus the results seen with a nontargeting control siRNA (Fig. 6B). However, in MEFs expressing ISG20, no detectable secretion of IFN-α/β (Fig. 6C) or upregulation of IFN genes (data not shown) was observed, demonstrating the role of ISG20 gene regulation in the absence of IFN production. Although the presence of IRF3 appears critical for ISG20 gene upregulation, analysis of IRF3 activation in the MEFs by assessment of phosphorylation at serine 396 or of nuclear migration of the protein by cell fractionation or antibody staining/confocal microscopy did not demonstrate activation/nuclear localization (data not shown). Together, these findings suggest that IRF3 contributes to the transcription profile observed with ISG20 expression; however, activation of the canonical pathway of IRF3 phosphorylation and nuclear migration is not observed.

### ISG20 inhibits replication of IFIT1-sensitive but not WT alphaviruses in mice.

Given the restriction observed with ectopic expression of ISG20 in MEFs, we sought to verify our findings in a mouse model of CHIKV infection and musculoskeletal disease (26–28). Wild-type C57BL/6 (B6) and *Isg20*^−/−^ male mice (3 weeks of age) were inoculated subcutaneously with 10^3^ PFU of CHIKV in the left rear footpad, and joint and tissue inflammation was tracked by direct measurement of swelling. Unexpectedly, we observed no differences in the levels of foot swelling between *Isg20*^−/−^ and B6 mice after CHIKV infection (Fig. S2A). Furthermore, serum, ipsilateral foot, and draining popliteal lymph node (PLN) samples from WT and *Isg20*^−/−^ mice showed no significant differences in viral RNA levels (Fig. S2B). In addition, serum IFN-α/β was not detectable in infected B6 and *Isg20*^−/−^ mice at 24 h.p.i. (data not shown).

10.1128/mSphere.00209-18.2 FIG S2 CHIKV pathogenesis is not altered in adult *Isg20*^−/−^ mice. Three-week-old male B6 or *Isg20*^−/−^ mice were infected subcutaneously with 10^3^ PFU of CHIKV. (A) Disease progression was assessed by footpad swelling, given by measurement of an elliptical cross-sectional area of the infected rear foot, over the course of disease. Data were normalized to the area measured on day 0 and are graphed as percentages of day 0 measurements. (B) Serum, footpad, and draining lymph node samples were collected at 24 h, and RNA was assessed for CHIKV genomes by qRT-PCR. (A) Results of 2-way ANOVA with Holm-Sidak *post hoc* analysis (*n* = 3, 2 independent experiments; data not statistically significant). (B) Mann-Whitney test results (*n* = 3, 2 independent experiments; data not statistically significant). Download FIG S2, TIF file, 0.3 MB. Copyright © 2018 Weiss et al. 2018 Weiss et al. This content is distributed under the terms of the Creative Commons Attribution 4.0 International license.

Neonatal pups lacking a fully developed immune system also can be used to assess differences in CHIKV pathogenesis between mouse strains through differences in survival times. Due to underdeveloped immune responses, loss of even a single ISG can result in susceptibility phenotypes (29, 30). *Isg20*^−/−^ and B6 pups were inoculated with 10^3^ PFU of CHIKV subcutaneously in the axial region and housed with a surrogate WT mother. Pups succumbing to disease earlier than 6 days p.i. manifested symptoms indicative of a systemic inflammatory response syndrome-like disease as observed with Sindbis virus (31). After 6 days, neurological signs developed consisting of ataxia progressing to hind limb paralysis. Notably, no differences in mean survival times or neurological disease manifestations were observed between *Isg20*^−/−^ and B6 neonatal mice infected with WT CHIKV (Fig. S3). Thus, although CHIKV infection is modulated by ectopic expression of ISG20 *in vitro* in MEFs, restriction in mice does not appear to be influenced by the absence of ISG20.

10.1128/mSphere.00209-18.3 FIG S3 ISG20 did not significantly impact survival in a lethal neonatal CHIKV challenge model. One-day-old B6 or *Isg20*^−/−^ pups were infected subcutaneously with 10^3^ PFU of CHIKV and tracked for (A) weight and (B) survival over the course of disease. Average time to paresis or paralysis is indicated in panel B, representing the only observable behavioral phenotype. Data represent results of log rank tests (*n* = 5 to 7, 2 independent experiments; data not statistically significant). Download FIG S3, TIF file, 0.4 MB. Copyright © 2018 Weiss et al. 2018 Weiss et al. This content is distributed under the terms of the Creative Commons Attribution 4.0 International license.

**FIG 7:**
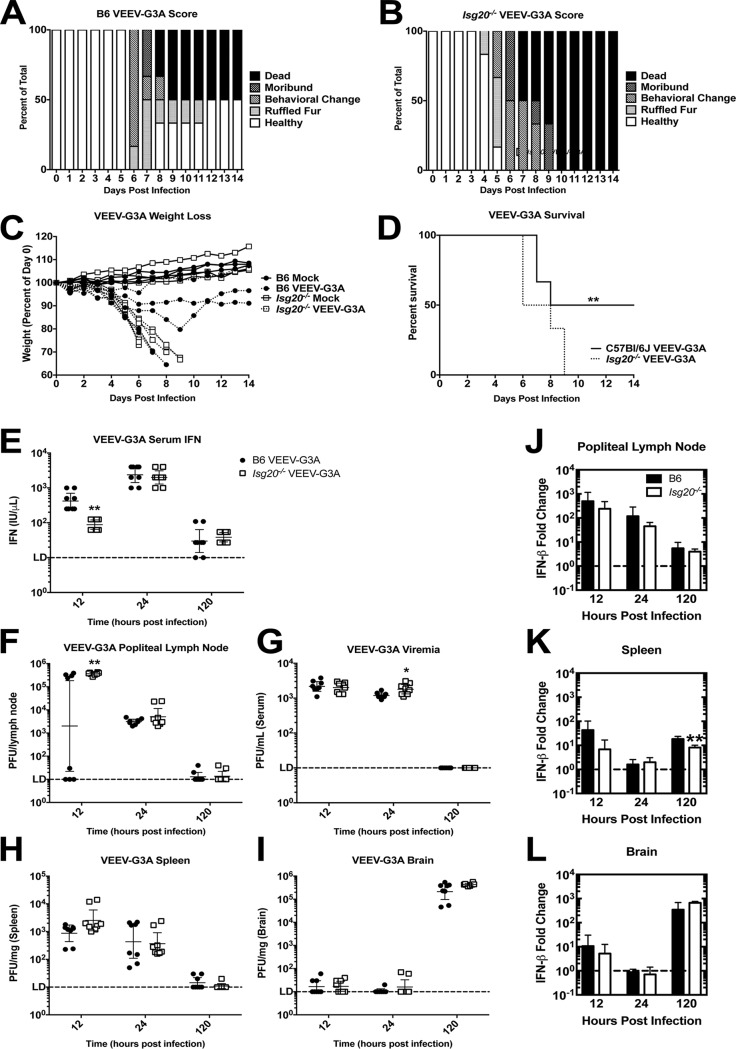
*Isg20*^−/−^ mouse data support the idea of increased virulence of the IFIT1-sensitive VEEV-G3A mutant. (A to D) B6 and *Isg20*^−/−^ mice received bilateral subcutaneous inoculations of 10^3^ PFU of VEEV-G3A in rear footpads and were monitored for signs of disease based on (A and B) clinical scoring of behavior, (C) weight loss, and (D) average survival. (E) Mice were sacrificed and perfused at the indicated time point, and tissues were assessed for serum IFN. (F to I) Virus replication was measured in (F) popliteal lymph node, (G) serum, (H) spleen, and (I) brain at the indicated time points. (J to L) IFN-β mRNA was measured in (J) popliteal lymph node, (K) spleen, and (L) brain after VEEV-G3A infection. Data are represented as fold change in IFN-β compared to mock treatment results. *, *P* < 0.05; **, *P* < 0.01. (D) log-rank test (*n* = 6 mice, 2 independent experiments). (E to I) Multiple Student’s *t* tests (*n* = 8 mice, 2 independent experiments). (F) Mann-Whitney test (*n* = 8 mice, 2 independent experiments).

IFIT1, which functions as an antiviral molecule that restricts virus translation (3, 6), was highly upregulated by ISG20 expression. The VEEV-G3A mutation disrupts the terminal stem-loop structure in 5′nontranslated regions, which allows IFIT1 recognition of the alphavirus type 0 cap structure, which is normally evaded by pathogenic strains (6). Age-matched, adult male mice were inoculated subcutaneously with 10^3^ PFU of VEEV WT or VEEV-G3A, and morbidity and mortality were monitored. VEEV WT-infected *Isg20*^−/−^ mice demonstrated a slightly earlier onset of symptoms, with clinical manifestations in B6 mice following about 24 h later (Fig. S4A and B). However, weight loss (Fig. S4C) and the median survival times for mice infected with VEEV WT were essentially equivalent at 5.5 and 5.8 days for B6 and *Isg20*^−/−^ mice (Fig. S4D). *Isg20*^−/−^ mice infected with VEEV-G3A displayed a worse disease score than B6 mice and developed neurological signs sooner than WT mice (Fig. 7A and B). Additionally, *Isg20*^−/−^ mice experienced more-rapid weight loss than WT mice after VEEV-G3A infection (Fig. 7C). Finally, *Isg20*^−/−^ mice uniformly succumbed to VEEV-G3A infection, whereas 50% of WT animals recovered by 12 days p.i. The median survival times after VEEV-G3A infection were 11 and 7 days in WT and *Isg20*^−/−^ mice, respectively (Fig. 7D).

10.1128/mSphere.00209-18.4 FIG S4 Knockout of ISG20 does not exacerbate VEEV-WT disease in mice. B6 and *Isg20*^−/−^ mice received bilateral subcutaneous inoculations of 10^3^ PFU of VEEV-WT (ZPC738) in rear footpads. Disease course was assessed by (A and B) clinical scoring specific to VEEV infection, (C) weight loss, and (D) survival rates. Data represent results of log rank tests (*n* = 3 to 4; data not statistically significant). Download FIG S4, TIF file, 0.6 MB. Copyright © 2018 Weiss et al. 2018 Weiss et al. This content is distributed under the terms of the Creative Commons Attribution 4.0 International license.

Innate immune responses within the first 24 h of infection often determine disease outcome, as the induction of the type I IFN response slows dissemination and primes the adaptive immune response. We measured VEEV-G3A burden and type I IFN levels in different tissues to determine the extent of virus replication and spread early (at 12 and 24 h.p.i.) and again at 5 days, when neurological symptoms typically appeared. At 12 h.p.i., we detected a 5-fold increase in serum IFN-α/β levels in B6 compared to the results seen with *Isg20*^−/−^ mice (*P* < 0.01). However, the serum IFN levels in *Isg20*^−/−^ mice increased to levels similar to those seen in B6 mice by 24 h.p.i. (Fig. 7E). VEEV-G3A infection in the draining popliteal lymph node (PLN) was elevated in *Isg20*^−/−^ mice compared to B6 mice at 12 h.p.i. (Fig. 7F). By 24 h.p.i., all WT mice had evidence of infection in their PLN, a time point at which time viremia was elevated ∼2-fold in *Isg20*^−/−^ mice (*P* < 0.05, Fig. 7F and G). By 5 days p.i., VEEV-G3A was undetectable in serum, PLN, or the spleen in both B6 and *Isg20*^−/−^ mice (Fig. 7F to H). Virus was measured at greater than 10^5^ PFU/mg in the brain at 5 days p.i. in B6 and *Isg20*^−/−^ mice, with the latter strain trending toward higher levels (Fig. 7I). No other significant differences in virus replication were detected in the spleen or the brain at any time point examined. IFN-β mRNA levels also were consistently lower in the PLN of *Isg20*^−/−^ mice than in that of B6 mice infected with VEEV-G3A, with fewer IFN-β transcripts also observed at 5 days p.i. in the spleen (Fig. 7K). IFN-β mRNA levels in the brain remained consistent between B6 and *Isg20*^−/−^ mice across all time points tested (Fig. 7J to L).

### Primary cells from Isg20^−/−^ mice are more susceptible to CHIKV and VEEV infections.

**FIG 8:**
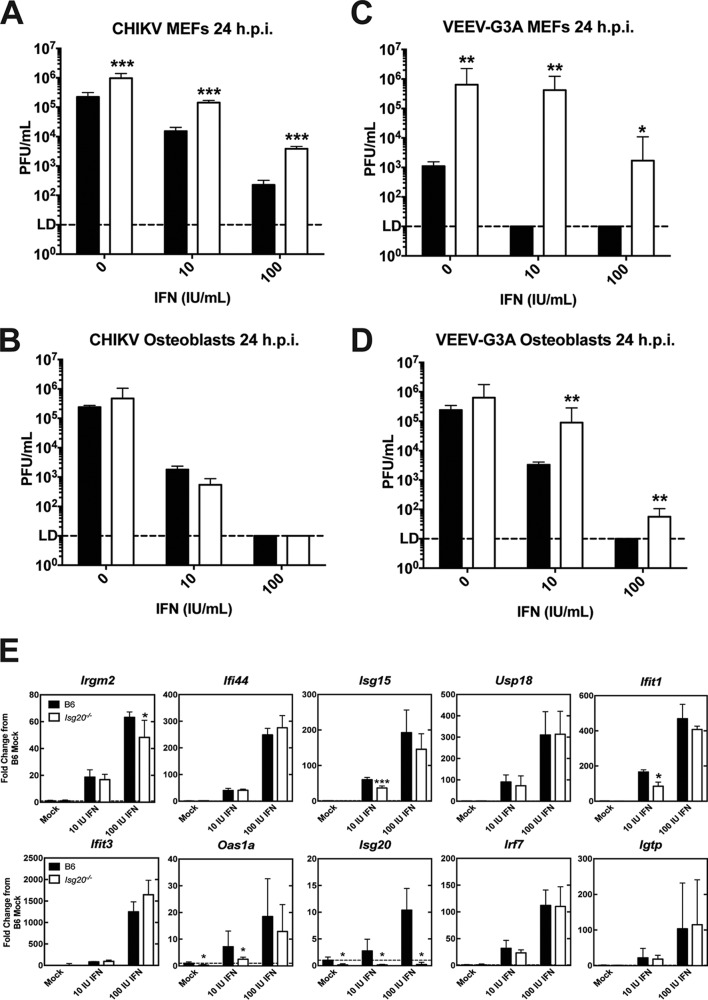
A deficiency of ISG20 leads to increased CHIKV and VEEV-G3A replication in primary cells. Primary cells were isolated from age- and sex-matched B6 and *Isg20*^−/−^ mice. Cells were treated with 0, 10, or 100 IU/ml IFN-α4/β (1:1 ratio) for 4 h and then infected with (A and B) CHIKV or (C and D) VEEV-G3A (MOI = 1). Virus titers were determined at 24 h.p.i. by plaque assay. (E) ISG induction was measured in uninfected, 6-h IFN-treated B6 or *Isg20*^−/−^ MEFs by qPCR. *, *P* < 0.05; **, *P* < 0.01; ***, *P* < 0.001 (multiple Student’s *t* tests). (A to D) *n* = 6, 2 independent experiments. (E and F) *n* = 3, 2 independent experiments.

The observed differences in viral replication suggest that ISG20 may participate in orchestrating innate immune responses in particular cell types, which affects the outcome of infection with IFIT1-sensitive viruses, which are less able to evade host antiviral responses. To begin to determine the cell-specific effects of an ISG20 deficiency, primary MEFs and osteoblasts were generated from sex- and age-matched wild-type and *Isg20*^−/−^mice; these cells represent possible early target cell types for CHIKV and VEEV *in vivo* (22, 32). In the absence of type I IFN priming, CHIKV replicated to ∼10-fold-higher levels by 24 h in *Isg20*^−/−^ MEFs than in WT MEFs (*P* < 0.01) (Fig. 8A). When MEFs were primed with 10 and 100 IU of IFN-α4/β at a 1:1 ratio for 4 h, we observed similar differences in virus replication, with ∼10-fold-greater infection in *Isg20*^−/−^MEFs than in B6 MEFs. In osteoblasts, only a minimal increase in CHIKV infection was detected in *Isg20*^−/−^ compared to B6 mice in the absence of IFN priming (Fig. 8B). After IFN priming, CHIKV replicated to similar levels in *Isg20*^−/−^and B6 osteoblasts, demonstrating cell-specific differences in ISG20-mediated antiviral effects (Fig. 8B). More-dramatic differences were observed with VEEV-G3A, as *Isg20*^−/−^ MEFs exhibited a 100-fold to 10,000-fold increase in VEEV-G3A infection compared to WT MEFs (*P* < 0.01) (Fig. 8C). These differences were maintained in the presence of IFN treatment, as B6 MEFs released no detectable virus at 24 h.p.i. (Fig. 8C). VEEV-G3A also replicated to higher levels after IFN treatment in *Isg20*^−/−^ primary osteoblasts than in B6 cells, albeit with 5-fold to 100-fold differences at 24 h.p.i. (*P* < 0.01) (Fig. 8D). The differences in primary cells indicate that ISG20-mediated gene regulation likely contributed to the antialphavirus profile and activity of ISGs. To determine whether IFN pretreatment of the primary B6 and *Isg20*^−/−^ MEFs led to the upregulation of antiviral ISGs, we measured the induction of the same genes that were upregulated in ISG20-overexpressing MEFs (Fig. 5). *Ifit1* induction was reduced by 2-fold in *Isg20*^−/−^ MEFs compared to B6 MEFs in response to low-dose (10 IU/ml) IFN treatment (*P* < 0.05) (Fig. 8E) but was abolished in response to higher-dose (100 IU/ml) IFN treatment. *Isg15* and *Oas1a* induction was also reduced in *Isg20*^−/−^ MEFs compared to B6 MEFs, and both are known antiviral ISGs that restrict virus replication (3, 33, 34).

## DISCUSSION

Type I IFN-stimulated genes have multiple roles in suppressing infection of many RNA and DNA viruses. Over 300 gene products can be induced by type I IFN, creating a broadly restrictive environment for both microbial and viral pathogens. The inhibitory activity of numerous ISGs has now been elucidated through extensive ectopic expression and short hairpin RNA (shRNA) profiling studies (3, 4, 35, 36), but the antiviral activity of many remains elusive. ISG20 was first biochemically characterized as an IRF1-induced gene with high homology to DEDD domain nucleases in the same family as the Saccharomyces cerevisiae *rex4p* gene (11, 37). ISG20 was demonstrated *in vitro* to have 3′–5′ exonuclease activity enabling nonspecific degradation of single-stranded nucleic acid substrates with a preference for RNA (11). Because mutations in the endonuclease catalytic domain abrogated antiviral activity, it has been hypothesized that ISG20 directly degrades viral RNA to achieve its antiviral effect (11, 13).

The antiviral activity of ISG20 in cell culture has been demonstrated in ectopic expression studies for a number of RNA viruses (3, 13–20), including the prototypical alphavirus, SINV (3). However, those studies did not define the mechanism of ISG20 restriction of replication in cells or animals. In the present study, we established a model system for determining the molecular basis of the restriction by murine ISG20. Stable ectopic expression of the dominant splice variant of murine ISG20 (isoform b), which shows 82% identity with human ISG20, inhibited CHIKV and VEEV replication *in vitro*, consistent with earlier findings determined with SINV. Introduction of a homologous glycine mutation at the D94 catalytic aspartate in the ExoII nuclease domain abrogated the antiviral activity of ISG20 *in vitro*, consistent with the published role of ISG20 catalytic activity in its antiviral function. However, in cells ectopically expressing the protein, the primary antiviral activity of ISG20 against alphaviruses occurred indirectly, through upregulation of subsets of IFN response genes.

Recent reports have suggested that ISG20 may mediate viral RNA degradation. Studies performed with hepatitis B virus (HBV) suggested that ISG20 restricts viral replication by directly degrading viral RNAs and genomic intermediates produced over the course of infection (20, 38). In our cell culture model, the 3′ terminus of the CHIKV genome did not undergo ISG20-mediated degradation, and viral RNAs were translation competent after exposure to the cytoplasm of ISG20-expressing cells, consistent with results obtained with IFN pretreatment of cells, which induces ISG20, among other cellular nucleases (24). Unlike HBV, the alphavirus RNA genome is replicated entirely in the cytoplasm and should not be exposed to the nuclear puncta that contain ISG20 and thus are not likely to be exposed to significant levels of ISG20 protein. These results are consistent with a previous study showing that ISG20 did not degrade or inhibit hepatitis C viral RNA, which is also localized in the cytoplasm (18).

Another study suggested that ISG20 directly interacts with influenza A viral proteins to block translation, binding specifically to the influenza virus nucleoprotein to facilitate this activity (19). One caveat with these experiments pertained to the abnormal distribution of ISG20 observed in a transient overexpression system, which favored a cytoplasmic localization, even in the absence of influenza virus nucleoprotein. In our experimental system, we observed the punctate, primarily nuclear localization of the tagged ISG20, consistent with previous studies (12). We also observed ISG20-specific restriction of virus RNA reporter translation. As translation restriction was not observed for reporter RNA containing an internal ribosomal entry site from EMCV or CrPV, it is likely that the block in translation is the result of the cap-dependent initiation mechanism and does not represent a general reduction in protein synthesis or mRNA stability. Notwithstanding these data, it is possible that ISG20 employs multiple mechanisms of translation restriction depending upon the virus. In light of recent work with HBV demonstrating ISG20 accumulation on particular secondary structures within viral RNAs, ISG20 may restrict nuclear replication of RNA viruses through direct binding and/or degradation (38). Indeed, HBV RNA binding by the catalytically inactive ISG20^D94G^ was preserved and contributed to an observed translation-suppressing phenotype (38). However, ISG20^D94G^ did not arrest translation of the alphavirus reporters, suggesting that direct RNA binding by ISG20 is not the primary mechanism of alphavirus translation suppression.

The localization of ISG20 may offer insight with respect to its mechanism of antiviral action. Despite its being a small, soluble protein capable of free diffusion through the nuclear pore complex, ISG20 localizes to the nucleus and interacts with the protein- and RNA-rich Cajal bodies (12). The primary purposes of these structures are the processing and modification of small nuclear RNAs that comprise the spliceosome (39, 40), suggesting that ISG20 may influence cytosolic virus replication through these additional mechanisms. In fact, it has been proposed that ISG20 could target the expression of host microRNA or long noncoding RNAs, which could regulate the expression of many different genes (38, 41). Our transcriptional analysis of MEFs ectopically expressing ISG20 revealed more than 100 upregulated genes that required the ISG20 exonuclease, including two interferon regulatory factor (*Irf*) genes, the cytoplasmic viral RNA-detecting helicase RIG-I gene (*Ddx58*), and genes encoding members of the IFN-induced protein with tetratricopeptide repeats (IFIT) family. *Ifit1* upregulation stands out in particular, because it inhibits translation of type 0 capped RNA in a manner consistent with our findings with respect to ISG20 expression (6); however, other ISGs could also contribute to the effects observed.

Among the alphaviruses, CHIKV is susceptible to murine type I IFN responses, and this effect has been attributed to numerous individual effector proteins (9, 30, 42–45). VEEV, by comparison, infects rodents as a reservoir host and has evolved mechanisms to evade the murine IFN response (46, 47). The VEEV-G3A mutant, with a single nucleotide mutation in the 5′ UTR, is attenuated in WT B6 mice, due, at least in part, to the action of *Ifit1*, an ISG that is also highly expressed in our cells overexpressing ISG20 (6). In our studies, VEEV-G3A was more virulent in the *Isg20*^−/−^ mice than in the WT B6 mice. In addition, lower levels of serum IFN-α/β were detected by 12 h in the *Isg20*^−/−^ mice concomitant with an increase in virus replication in the lymph node. The attenuation of the VEEV-G3A mutant was previously shown to be highly dependent on IFN-α/β responses (48) and also on IFIT1 (6) specifically. Additionally, the tested *Isg20*^−/−^ primary cells showed increased susceptibility to alphavirus infection, even in the presence of IFN priming. The reduced induction of *Ifit1*, *Isg15, and Oas1a* mRNA levels that we observed in the primary *Isg20*^−/−^ MEFs compared to B6 MEFs after IFN treatment may be sufficient for VEEV-G3A escape of IFIT1-mediated repression. However, *in vivo*, the increased susceptibility of *Isg20*^−/−^ mice to VEEV-G3A infection may also be due to interferon induction differences, which may or may not be related to differences in upregulation of the specific ISGs identified in our *in vitro* screens. Combined with our understanding of IFN-mediated ISG20 upregulation and ISG20-mediated upregulation of IFIT1 and other antiviral effectors *in vitro*, the mouse model of VEEV-G3A suggests a feed-forward mechanism by which ISG20 amplifies IFN and ISG production in response to infection. The failure of the ISG20-expressing fibroblasts to produce IFN-α/β may reflect cell type-dependent differences in the effect of ISG20.

We propose a model where expression of ISG20 blocks incoming alphavirus genome translation and possibly other replication activities through the modulation of antiviral ISGs. This modulation of the host antiviral environment is achieved through a direct upregulation of ISGs possibly involving IRF3 and, *in vivo*, augmented by a positive loop of IFN production. Future studies should elucidate the extent to which IFIT1 is responsible for alphavirus translation inhibition and whether alternative mechanisms are at play, as well as whether or not a noncanonical activity of IRF3 is involved in mediating the ISG20 signal.

## MATERIALS AND METHODS

### tet-off MEF cells.

tet-off murine embryonic fibroblasts (MEFs) expressing ISG20, enhanced green fluorescent protein (GFP) control, or firefly luciferase (fLuc) control genes were previously described, and tet-off MEFs expressing ISG20^D94G^ (ExoII) cells were generated similarly (3). The ExoII gene was made by aligning the human and mouse sequences and mutating the active site of the exonuclease to match the human ExoII nuclease-deficient mutant (13). tet-off MEFs were maintained in complete media consisting of Dulbecco’s modified Eagle’s medium (DMEM) supplemented with 10% heat-inactivated fetal bovine serum (FBS), 200 mM l-glutamine, 10,000 units/ml penicillin, 10 mg/ml streptomycin, 100 μg/ml G418 sulfate, and 100 μg/ml hygromycin sulfate. Gene overexpression was suppressed with the addition of 2 μg/ml doxycycline hyclate (Sigma) for cell expansion prior to experiments.

### Ectopic expression of target genes.

tet-off MEFs were trypsinized and washed in sterile phosphate-buffered saline (PBS) three times, plated, and induced in doxycycline-free, complete tet-off MEF media for 72 h prior to use. Gene expression was confirmed by both qRT-PCR and Western blotting for these conditions. All cells were grown to approximately 80% confluence for optimal infection and transfection conditions in subsequent experiments.

### Western blotting.

MEFs were induced for 72 h as described above and then lysed in radio immunoprecipitation assay buffer with protease and phosphatase inhibitors (2). Cell wall debris was cleared by centrifugation, and 20 ng of protein was electrophoresed on 5%/10% SDS polyacrylamide discontinuous gels. Gels were transferred to a polyvinylidene difluoride (PVDF) immunoblotting membrane by semidry transfer and blocked with 5% nonfat milk–Tris-buffered saline–0.1% Tween 20 (TBS-T) for 1 h. Anti-FLAG M2-peroxidase conjugate (1:2,000) was applied for 2 h at room temperature, or rabbit anti-mIfit1 (1:1,000) was applied overnight at 4°C with agitation. Secondary detection of IFIT1 was performed by thoroughly washing in TBS-T followed by goat anti-rabbit horseradish peroxidase (HRP) conjugate (1:2,000) for 1 h at room temperature. Blots were thoroughly washed prior to detection with Pierce ECL detection reagent (Thermo Scientific) and exposure to chemiluminescence film (GE Healthcare).

### Viruses.

The wild-type CHIKV la Réunion (CHIKV-LR) cDNA clone (49) was a gift from Stephen Higgs (Kansas State University). The WT VEEV (ZPC738) and VEEV-G3A cDNA clones were published previously (48, 50). eGFP or nanoluciferase (nLuc) was inserted into CHIKV or VEEV cDNA clones either in nsP3 nonstructural protein or in the structural proteins as a self-cleavable protein (*Thosea asigna* virus [TaV]) as previously described (21). Viruses were generated from infectious cDNA clones after *in vitro* transcription with SP6 polymerase (mMessage mMachine; Ambion) and electroporation in BHK-21 cells. Supernatants were collected at 24 h and centrifuged to remove cell debris prior to freezing in aliquots at −80°C. All viral titers were determined by BHK-21 plaque assay and are expressed in PFU per milliliter.

### Virus growth assays.

A 50-μl volume of supernatant was collected from infected cells at various times postinfection, and virus titers were determined by plaque assay on BHK-21 cells. nLuc reporter-virus infected cells were washed 3 times in PBS, lysed in luciferase passive lysis buffer, and then frozen to aid in complete disruption of cellular membranes. A 25-μl volume of each sample lysate was combined with 25 μl of prepared NanoGLO chemiluminescent reagent and incubated for 6 min at room temperature. Chemiluminescent signal was detected on a luminometer, quantified as relative light units, and normalized to individual sample protein concentrations determined by bicinchoninic acid (BCA) protein assay.

### Immunofluorescence microscopy.

Cells grown on glass coverslips were fixed in 4% paraformaldehyde for 15 min and permeabilized with 0.1% Triton X-100–PBS for 15 min. Fixed cells were rehydrated in PBS–0.5% BSA (PBS-B) and blocked for 45 min in 20% serum–PBS-B, corresponding to the secondary detection antibody species. Primary and secondary detection antibodies were applied for 1 h each at room temperature in PBS-B. Immunofluorescence was preserved with SlowFade Gold antifade reagent with DAPI (4′,6-diamidino-2-phenylindole; Invitrogen) and mounted on glass slides for confocal or epifluorescence microscopy. M2 anti-FLAG fluorescein isothiocyanate (FITC) conjugate antibody (Sigma) was used for detection of FLAG-tagged murine ISG20 and IG20^D94G^. Percentages of infected cells were calculated by counting the number of GFP cells/total number of DAPI nuclei in the field.

### Translation reporters and double electroporation.

The nonreplicating host, EMCV, and CHIKV translation reporters were produced as described previously (2, 51). The cricket paralysis virus (CrPV) translation reporter, a gift from Martin Bushell, Medical Research Council, United Kingdom, was described previously (52). tet-off MEFs were induced on 150-mm-diameter dishes as described above for ectopic expression of the gene of interest. Induced cells were trypsinized and washed once in Opti-MEM. Approximately 3 × 10^7^ cells were resuspended in 1 ml of Opti-MEM per reaction and electroporated with 7.5 μg of the indicated reporter RNA and 750 ng of *Renilla* luciferase mRNA using a Bio-Rad GenePulser (220 V, 1,000 μF, 2 pulses). Electroporated cells were diluted in doxycycline-free media and divided into 96-well plates. Cells were collected at the indicated time points by centrifugation, washed once in PBS, and lysed in luciferase passive lysis buffer. A 25-μl volume of collected lysates was assayed by dual-luciferase assay (Promega) and normalized to protein concentrations as determined by BCA protein assay (Thermo Scientific). For the double-electroporation experiment, MEFs were electroporated with 7 μg of the CHIKV translation reporter as described above. Cells were washed in PBS and incubated in doxycycline-free media for 0 h or 3 h. At each time point, cells were washed with PBS and added to Tri reagent (Molecular Research Center). RNA was extracted according to the manufacturer’s guidelines and resuspended in Opti-MEM. The freshly isolated RNA was then reelectroporated into MEFs expressing either eGFP or ISG20 and incubated for 3 h in doxycycline-free media. At 3 h, lysates were collected for luciferase analysis using a firefly luciferase assay system (Promega). Luciferase activity arising from the second transfection is given as a ratio of firefly luciferase signal from 3 h versus 0 h samples corresponding to the first transfection.

### Cycloheximide treatment of tet-off MEFs.

For cycloheximide treatment, GFP, ISG20, or ExoII tet-off MEFs were infected with CHIKV (MOI = 5) expressing nLuc in frame with the nsP3 nonstructural protein for 1 h. After washing, medium containing either 0 or 10 μM of cycloheximide (Sigma) was added for the duration of the experiment to block translation. At indicated time points, cells were washed with PBS and lysed with 1× passive lysis buffer (Promega). A 25-μl volume of collected lysates was assayed by Nano-Glo luciferase assay (Promega) and normalized to protein concentrations as determined by BCA protein assay (Thermo Scientific). Data are represented as relative luminescence units per microgram of protein (RLU/μg). For RNA degradation experiments, MEFs were infected with CHIKV-LR (MOI = 0.1) and treated with 10 μM cycloheximide. At the indicated time points, the cells were washed and placed in Tri reagent (Molecular Research Center) according to manufacturers’ guidelines. cDNA was reverse transcribed using random hexamers from 100 ng of Tri reagent-extracted RNA and detected by quantitative PCR (qPCR) with SYBR green on a MiniOpticon thermal cycler and detection unit (Bio-Rad) using the following primers: CHIKV-3′NTR-F (5′-ATA ATT GGC AAA CGG AAG AGA T-3′) and CHIKV-3′NTR-R (5′-ACA AAA TAA CAT CTC CTA CGT CC-3′).

### RNA sequencing.

Total cellular RNA from two separately derived clones of tet-off MEFs expressing eGFP, Isg20, or ISG20^D94G^ was isolated and depleted of rRNA with Ambion RiboMinus Eukaryote kit v2 (Life Technologies). Directional sequencing libraries were generated using an NEBNext Ultra Directional RNA Library Prep kit for Illumina with NEBNext Primer Set 1 (New England BioLabs). Multiplexed libraries were sequenced with 100-bp paired-end reads on an Illumina HiSeq 2000 platform (Axeq Technologies). Reads were aligned to the iGenome-indexed Mus musculus genome UCSC mm9 (Illumina) using Tophat, and differential gene expression was determined using Cufflinks (53). Pathway analysis of differentially regulated genes was performed using Ingenuity pathway analysis (Qiagen).

### qRT-PCR.

Forward and reverse primers for qRT-PCR were designed as follows: for *Isg20*-F, 5′-AAC ATC CAG AAC AAC TGG CGG-3′; for *Isg20*-R, 5′-GTC TGA CGT CCC AGG GCA-3′; for *Irgm2*-F, 5′-GCG ATA GAG ATT CGG AAA GC-3′; for *Irgm2*-R, 5′-CAG CAC CCA GTC ATC TTG TT-3′; for *Usp18*-F, 5′-AGG AGT CCC TGA TTT GCG TG-3′; for *Usp18-*R, 5′-GGG TTT TCA GAG GCT TTG CG-3′; for *Ifit3*-F, 5′-AGA TTT CTG AAC TGC TCA GCC C-3′; for *Ifit3*-R, 5′-CAG AGA TTC CCG GTT GAC CTC-3′; for *Irf7*-F, 5′-ATT TCG GTC GTA GGG ATC TG-3′; for *Irf7*-R, 5′-GTT GGT CTT CCA GCC TCT TC-3′; for *Ifi44*-F, 5′-ACT CGT TTG ACA TGG CAG CA-3′; for *Ifi44*-R, 5′-TCT GCA CAC TCG CCT TGT AA-3′; for *Ifit1*-F, 5′-GTG GCT CAC ATA GAG CAG GA-3′; for *Ifit1*-R, 5′-AGT TTC CTC CAA GCA AAG GA-3′; for *Oas1a*-F, 5′-TCC ACA GTA CGC CCT AGA GT-3′; for *Oas1a*-R, 5′-GAC CAG TTC CAA GAC GGT CC-3′; for *Igtp-*F, 5′-TCT GAG CAG GTT CTG AAG GA-3′; for *Igtp*-R, 5′-TCC TCG GCT TCT TTC TTC TC-3′; for *Isg15*-F, 5′-TCC ATG ACG GTG TCA GAA CT-3′; for *Isg15-*R, 5′-GAC CCA GAC TGG AAA GGG TA-3′; for *Ifit2*-F, 5′-AGA ATT CAC CTC TGG ATG GG-3′; for *Ifit2*-R, 5′-GTC AAG CTT CAG TGC CAA GA-3′; for *Irf3*-F, 5′-GCG GTT AGC TGC TGA CAA TA-3′; for *Irf3*-R, 5′-AGG CCA TCA AAT AAC TTC GG-3′, for *18S*-F, 5′ CGC CGC TAG AGG TGA ATT TCT-3′; and for *18S*-R, 5′ CGA ACC TCC GAC TTT CGT TCT-3′. qRT-PCR primers for CHIKV positive-strand RNA detection were as previously described (54). cDNA was reverse transcribed with random hexamers from 100 ng of Tri reagent-extracted RNA and detected by qPCR with SYBR green on a MiniOpticon thermal cycler and detection unit (Bio-Rad). Fold induction was determined for genes of interest using the cycle threshold (ΔΔ*C_T_*) method.

### Promoter activation reporters.

Construction of promoter luciferase plasmids pRL-SV40, pβLUX (a gift from Barbara Sherry, North Carolina State University) (55), and PRDI/III or PRDII (gifts from Tom Maniatis, Harvard University) (56) was described previously. GFP, ISG20, and ExoII MEFs were induced in 24-well cluster plates for expression of the target protein for 3 days as described above. Promoter luciferase plasmid (0.5 μg/well) and simian virus 40 (SV40) promoter-driven *Renilla* luciferase control plasmid (0.25 μg/well) were transfected into MEFs using TransIT-LT1 reagent (Mirus) and incubated for 24 h. Cells were treated 16 h prior to harvest with 0.3 μg poly(I·C)–TransIT-LT1 reagent or transfection reagent alone. Lysates were collected in passive lysis buffer and measured by dual-luciferase assay (Promega). Firefly luciferase RLUs were normalized to *Renilla* RLUs in each cell and are represented as percentages of untreated GFP control cells.

### siRNA knockdown of IRF3 in MEFs.

GFP, ISG20, and ExoII MEFs were induced as described above. After 24 h, 25 nm of SMARTpool siRNA *Irf3* (Dharmacon) or 25 nm of SMARTpool nontargeting siRNA (Dharmacon) was transfected into MEFs using DharmaFECT 1 transfection reagent according to manufacturer’s guidelines. After 48 h, cells were washed and used for RT-PCR analysis of indicated genes. Data were normalized to 18S values, and fold change compared to sham-treated control GFP cell results is graphed.

### Mice and infections.

Male or pregnant female C57BL/6J (B6) mice were purchased from the Jackson Laboratory. *Isg20*^−/−^ mice were generated at Washington University after receiving heterozygous *Isg20*^+/−^ sperm from C57BL/6 mice containing a promoter knockout [*Isg20^tm1a^*^(^*^KOMP^*^)^*^Wtsi^*] from the Knockout Mouse Project Repository (KOMP; University of California, Davis). The sperm was used for *in vitro* fertilization of eggs from B6 recipient female mice. Heterozygous *Isg20*^+/−^ mice were backcrossed to establish the *Isg20*^−/−^ colony. *Isg20*^−/−^ mice produced normal litter sizes of expected Mendelian ratios, with all progeny appearing healthy. Mice were used at 1 day or 3 weeks of age for CHIKV infection and at 6 weeks of age for VEEV infection. CHIKV (10^3^ PFU) was inoculated subcutaneously in the left rear footpad in 10 μl of Opti-MEM. VEEV was inoculated subcutaneously in each rear footpad with 10^3^ PFU for a total of 2 × 10^3^ PFU per animal. Disease was monitored by changes in weight and clinical scoring specific to the disease manifestations of each virus every 12 to 24 h. For virus titration, RNA isolation, and IFN bioassay, serum was collected from submandibular vein, and mice were euthanized and perfused with 10 ml of PBS before tissue collection. Tissues were collected in 100 μl of PBS–1% bovine serum per gram of tissue and mechanically dissociated, and virus titers were determined by BHK-21 plaque assay performed on the resulting supernatants. Serum was assayed for functional type I IFN using a bioassay as previously described (57). All animal experiments were conducted under the guidance of protocols approved by the Institutional Animal Care and Use Committee of the University of Pittsburgh.

### Generation and infection of primary cells.

Osteoblasts were prepared by dissecting calvaria from 4-day-old pups and manually removing surrounding tissue. Calvaria were washed in PBS and digested in two 20-min digestions and one 90-min digestion in α-MEM with 96 μg/ml collagenase P and 0.01% trypsin–EDTA on a shaking 37°C incubator. Digested calvaria were washed in PBS and suspended in α-MEM supplemented with 10% FBS, 10,000 units/ml penicillin, and 10 mg/ml streptomycin for 5 days undisturbed on 100-mm-diameter cell culture dishes. Osteoblasts were trypsinized and expanded for two passages on T-75 flasks prior to infections.

Primary MEFs were prepared from pregnant mice at 14 days of gestation. The head and liver was removed from individual embryos, and specimens were rinsed in PBS. Embryos were minced in ice-cold 0.25% trypsin–EDTA solution and then heated to 37°C for 30 min in a water bath. MEFs were homogenized by serial passage through 18- and 23-gauge needles. Cells were washed and resuspended in DMEM supplemented with 10% heat-inactivated FBS, 10,000 units/ml penicillin, and 10 mg/ml streptomycin. MEFs were expanded to passage 2 for individual experiments. For infection, cells were treated with 0, 10, or 100 IU/ml of IFN-α4/β (1:1 ratio) for 4 h, washed, and then infected with either CHIKV or VEEV-G3A at an MOI of 1 for 1 h. The cells were washed thrice with PBS, and medium specific for each cell type was added. Virus was collected at 24 h.p.i., and titers were determined by plaque assay on BHK-21 cells. For RT-PCR, primary B6 or *Isg20*^−/−^ MEFs were treated with 0, 10, or 100 IU/ml of IFN-α4/β (1:1 ratio) for 6 h, washed with PBS, and placed in Tri reagent for RNA isolation and qRT-PCR for indicated genes.

### Statistical analysis.

The statistical model for differential gene expression by RNA-seq was described in detail previously (58). All other statistical analyses were performed in GraphPad PRISM. Analysis of variance (ANOVA) was performed with Dunnett’s multiple-comparison test. Two-way ANOVA was performed with the Holm-Sidak multiple-comparison test. Two-tailed Mann-Whitney and Student’s *t* tests were performed for α = 0.05.

### Data availability.

The RNA sequencing data have been deposited in the Gene Expression Omnibus (GEO) database with accession number GSE115729.
