# Supplementary figures and images for: The Interferon-Induced Exonuclease ISG20 Exerts Antiviral Activity through Upregulation of Type I Interferon Response Proteins
Source: mSphere. 2018 Sep 19;3(5):e00209-18. doi: 10.1128/mSphere.00209-18 (PMC6147134; doi:10.1128/mSphere.00209-18)

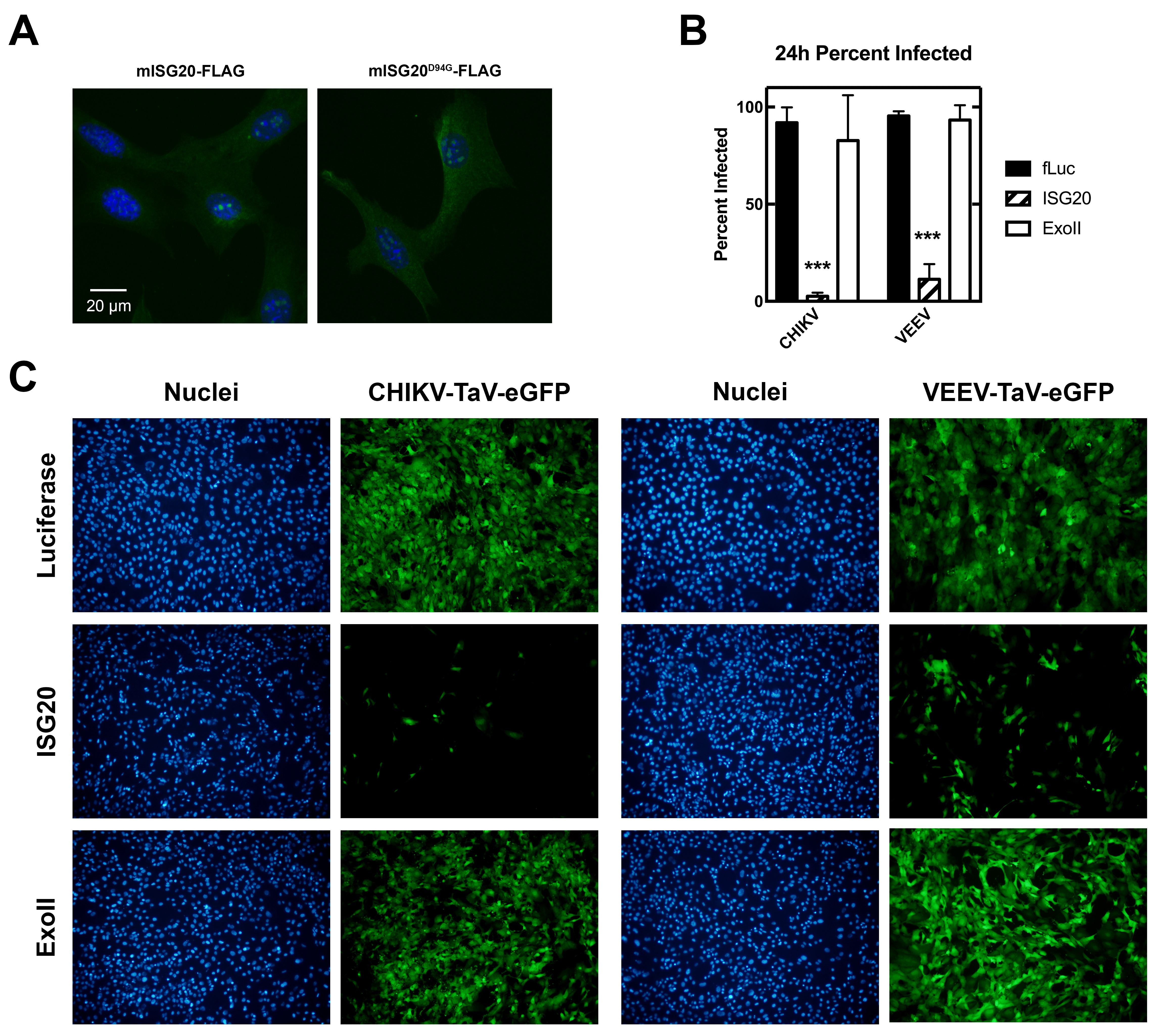

Supplement: FIG S1 [file sph005182641sf1.jpg]

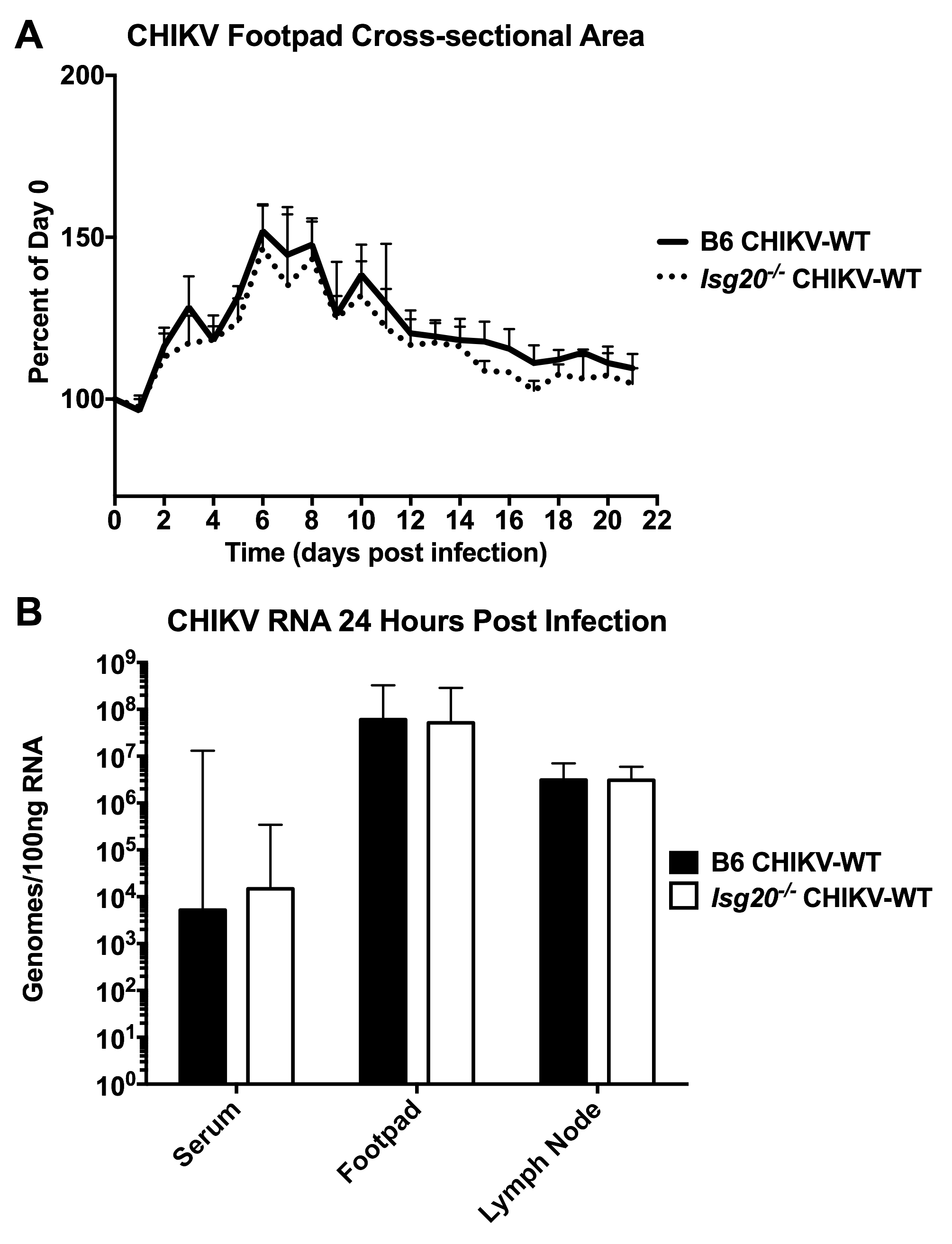

Supplement: FIG S2 [file sph005182641sf2.tif]

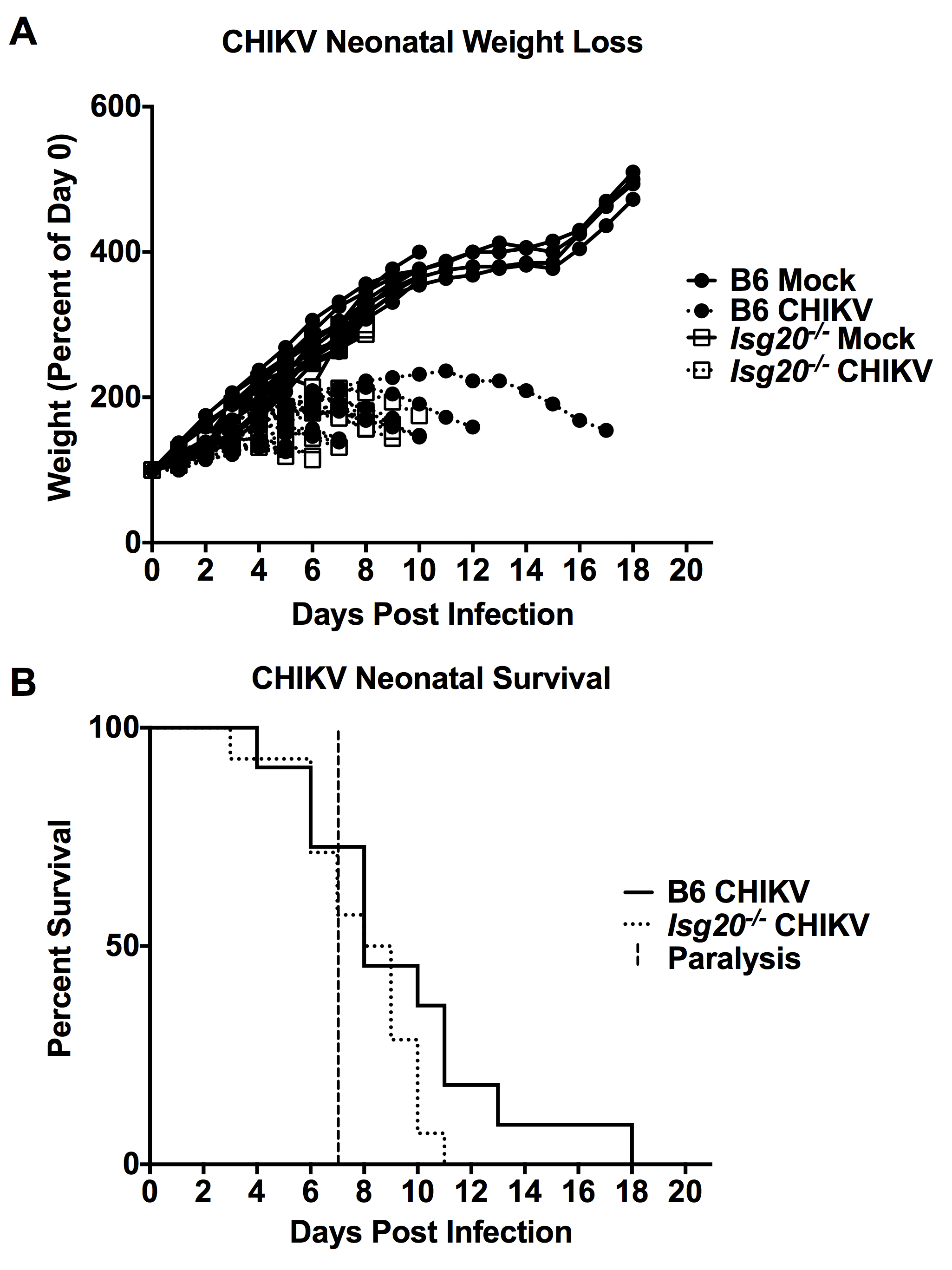

Supplement: FIG S3 [file sph005182641sf3.tif]

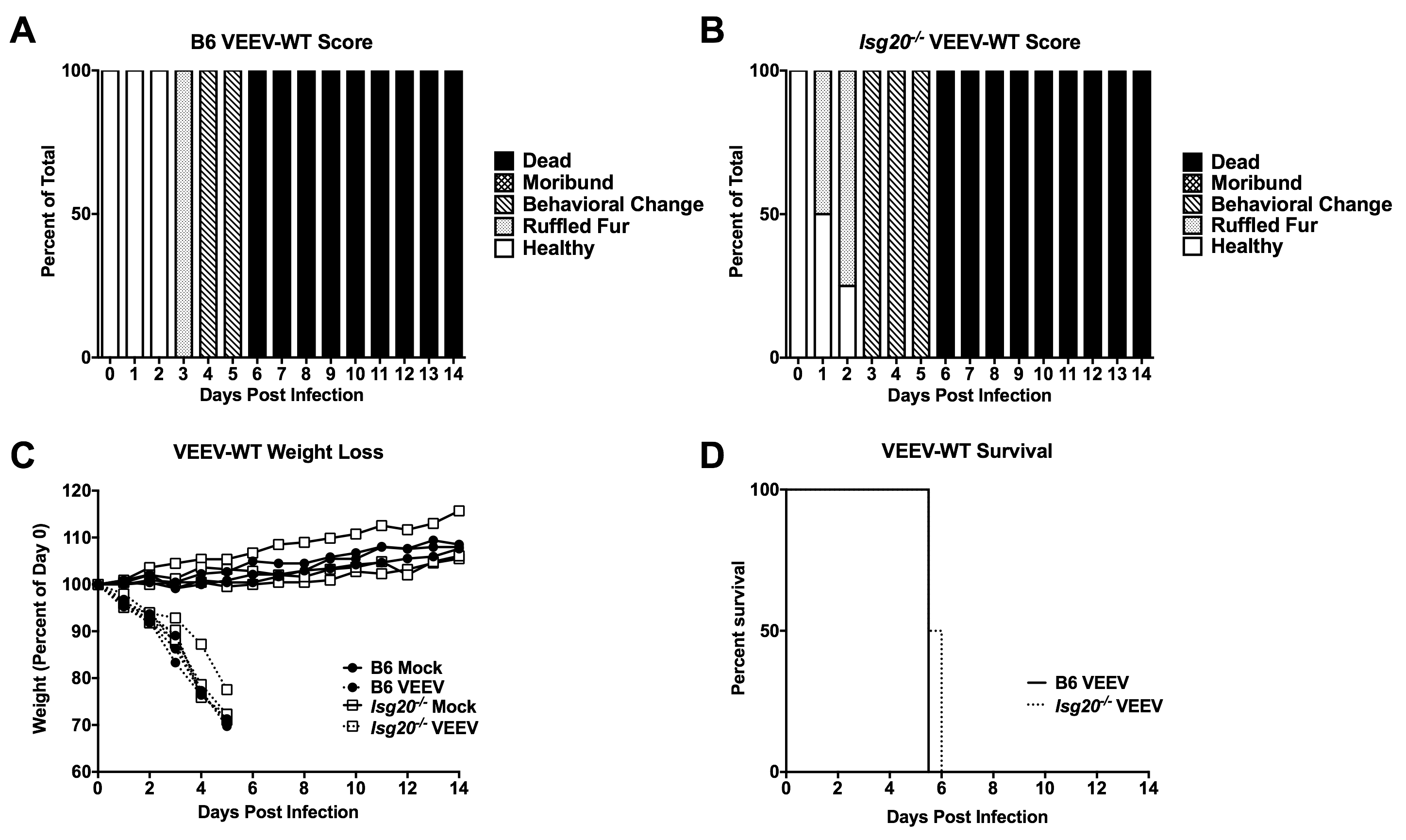

Supplement: FIG S4 [file sph005182641sf4.tif]
